# Maternal work and exclusive breastfeeding practice: a community based cross-sectional study in Efutu Municipal, Ghana

**DOI:** 10.1186/s13006-017-0100-6

**Published:** 2017-02-10

**Authors:** Jacqueline Nkrumah

**Affiliations:** Department of Health Administration and Education, Faculty of Science Education, University of Education, Winneba, Ghana

**Keywords:** Exclusive breastfeeding, Breastfeeding frequency, Maternal work, Formal and informal employment

## Abstract

**Background:**

Maternal work has been identified as one of the factors that affect exclusive breastfeeding in the first six months of life. In Ghana, mothers engaged in the formal sector of employment are unable to exclusively breastfeed after maternity leave because facilities at their work places and conditions of work do not support exclusive breastfeeding. Even though maternal work and exclusive breastfeeding does not seem well matched, not all maternal work are incompatible with the practice of exclusive breastfeeding.

This study seeks to identify the features of maternal work associated with exclusive breastfeeding in Effutu Municipal in the Central Region of Ghana. To achieve the above aim, I discuss the different types of maternal work, their characteristics, and how the work may influence exclusive breastfeeding.

**Methods:**

The study is a community based cross-sectional study involving 225 mother-infant pairs attending community based Child Welfare Clinics in Effutu Municipal, Ghana. Data were collected through face-to-face structured interviews and focus group discussions over a period of five months in 2015. Data on breastfeeding is based on the breastfeeding practice in the 24 h preceding the survey. Chi-square test is used to identify strength of association between the features of maternal work and exclusive breastfeeding practice.

**Results:**

The exclusive breastfeeding rate among mothers with infants between the ages of 0 – 5 months is 72%. The mean and median number of breastfeeds per day is 11 ± 2.7 and 13.5 respectively. A significant difference in exclusive breastfeeding was established between mothers in the formal (16%) and informal (84%) sectors of employment (*p* = 0.020). The study also established a significant difference in breastfeeding frequency between mothers in the formal (9%) and informal (91%) sectors of employment (*p* = 0.001). There was also a significant difference in breastfeeding frequency among respondents that go to work with their infant (64%) and those who do not go to work with their infant (36%) (*p* = 0.000).

**Conclusion:**

Interventions to promote exclusive breastfeeding should include the use of existing family structures, supportive cultural beliefs, and practices and promotion of an infant-friendly work environment.

## Background

The promotion of exclusive breastfeeding is the most cost-effective intervention among all the interventions to reduce infant mortality in developing countries [1, 2]. Breastfeeding provides infants with the ideal nourishment needed for growth and development. It has the appropriate quantities of nutrients in breast milk which can easily be digested and protect both the mother and child against illness and diseases [3].

According to Galipeau and colleagues, breastfeeding frequency impacts the initiation of lactogenesis II, which was found to influence duration of exclusive breastfeeding [4]. Based on Galipeau and colleagues findings, they concluded that breastfeeding should be done as frequent as eight to twelve times a day [4]. In Ghana, the breastfeeding policy advocates that mother’s breastfeed at least eight to twelve times per 24 h period to ensure that babies receive enough milk [5].

Breastfeeding was identified as a practical intervention for achieving the Millennium Development Goal (MDG 4) of reducing overall child mortality by 2015 in Ghana [6]. Breastfeeding promotion in Ghana is an aspect of Child Health Program. It is under the domain of the Reproductive and Child Health (RCH) unit of the Ghana Health Service (GHS) [7]. This unit is duplicated in all public health facilities across the country. Exclusive breastfeeding is promoted for the first six months of life of the infant and breastfeeding continues until two years of age, in addition to the introduction of appropriate complementary feeding.

Prior to the introduction of the MDG’s, several interventions were introduced to promote breastfeeding practice in Ghana; prominent among them was the Baby Friendly Hospital Initiative (BFHI) introduced in 1993 and re-inaugurated in February 2013 with the nomenclature “Baby Friendly Hospital Initiative Authority” (BFHIA) to oversee the implementation of exclusive breastfeeding policies and interventions in Ghana [8].

As part of Child Health Program of the Ghana Health Service, exclusive and continuous breastfeeding education and counseling are integrated into Adolescent, Antenatal, Delivery and Postnatal care, as well as Child Welfare Clinics (CWC’s). All expectant mothers who contact health facilities receive education and counseling on exclusive and continuous breastfeeding during pregnancy and after delivery [7]. The Baby Friendly Hospital Initiative also ensures that all mothers who deliver in health facilities are trained on how to sit and correctly position their babies for breastfeeding. The purpose of these practices is to ensure that the majority of pregnant women are well informed on the importance and benefit of breastfeeding to their health and that of their babies before delivery.

There are also in place legislative provisions that support exclusive breastfeeding in the early life of infants. Ghana is among countries that have implemented the provisions of the International Breastfeeding Code on Marketing of Breast Milk Substitute through legislation. That is, the Breastfeeding Promotion Regulation Act 2000 (LI 1667) and there is also in place, measures for monitoring the provisions of the Code on a regular basis [9].

Article 57 (1) of Act 651 of 2003 provides paid maternity leave for a period not less than twelve weeks upon the production of a medical certificate issued by a medical practitioner or a midwife indicating the expected date of confinement of women in formal employment in addition to their annual leave. Added to this, the Act also makes provision of a one hour break for nursing mothers to breastfeed their babies during working hours. This hour break is treated as part of working hours and paid for accordingly [10].

The problem with breastfeeding practice in Ghana is that the majority of Ghanaian mothers partly breastfeed their infants, and fail to make the most of the many benefits of breast milk [11]. Ghana recorded 61% improvement in exclusive breastfeeding from 1992 – 2003 [6]. However, the report of the 2011 Multiple Indicator Cluster Survey (MICS) indicates that exclusive breastfeeding has reduced from 63% in 2003 to 46% in 2011 [12]. It is important to investigate the factors that affect the practice of exclusive breastfeeding so that appropriate interventions can be put in place to improve the practice.

Even though some provisions in the Labor Act support breastfeeding, it has been revealed that the provisions are not adequate enough to support mothers who wishes to continue exclusive breastfeeding after resuming duty from maternity leave [13, 14]. This study found that some work places in Ghana do not have facilities that support working mothers to continue exclusive breastfeeding after maternity leave. Anecdotal evidence in Ghana also shows that women in certain occupation and/or profession are not permitted to be at work with their babies. This situation to a large extent discourage mothers from exclusively breastfeeding their infants as recommended by the World Health Organization (WHO).

Huffman has suggested that work location, type of work and income derived from work are some of the factors that influence the relationship between maternal employment and breastfeeding prevalence [15].

Another study by DaVanzo and Lee revealed that the type of work mothers do significantly affects whether they will remain with their children or not [16]. In this study, it was found that women engaged in work such as dressmaking, food and beverage making, farming or weaving had their children less than ten years of age with them, compared to those in formal employment. This suggests that certain types of work provide an opportunity for mothers to be with their children while working. Such mothers are more likely to exclusively breastfeed and increase their feeding frequency.

Recent studies on determinants of exclusive breastfeeding mention maternal employment as one factor that influences the practice [17–19]. In Ghana, studies on maternal work and exclusive breastfeeding in parts of the country have looked into exclusive breastfeeding practice of formal sector working mothers, and have found that mothers in this sector are unable to practice exclusive breastfeeding as recommended by the WHO due to conditions prevalent at their work places. These studies found that exclusive breastfeeding rate among formal sector working mothers ranges from 10% to 50% depending on the type of work [20, 21]. However, studies on mothers in the informal sector of employment and exclusive breastfeeding have not been explored.

The above studies did not consider the features of maternal work associated with exclusive breastfeeding practice. Therefore, this study investigates exclusive breastfeeding and its association with the features of maternal work and exclusive breastfeeding practice in the Effutu Municipality in the Central Region of Ghana. The study also takes a critical look at breastfeeding frequency because the Ghana Breastfeeding Program and Child Health Policy promote breastfeeding on demand. However, to ensure that babies receive enough milk, emphasis is placed on breastfeeding at least 8 to 12 times per 24 h period [5]. Feeding on demand and at least 8 to 12 times per 24 h is advocated at antenatal, postnatal and Child Welfare Clinics in Ghana. It has also been said that the physiologic process of milk production and output is dependent on the suckling process, which includes frequency, intensity and duration [5, 15].

Since breast milk production to some extent depends on breastfeeding frequency, and the duration of exclusive breastfeeding also depends on milk output, it is important that we consider breastfeeding frequency when looking at the association between the features of maternal work and exclusive breastfeeding.

## Methods

### Study area and participants

The study was conducted from July to November, 2015 in Effutu Municipality in the Central Region of Ghana. Effutu municipality is one of the twenty districts in the Central Region, and is about 66 km way from the capital city of Ghana (Accra). It has a projected population of 75,117 from the 2010 population census with a growth rate of 2.3% per annum and a population of women in fertility age and children aged less than one year at 17,844 and 3,103 respectively [22]. Administratively, the municipality is divided into four sub-areas called sub-municipalities; namely, South-East Winneba, South-West Winneba, Essuekyr-Gyahadze and Kojo-Bedu North/Low Cost Sub-Municipalities.

The municipal health system is organized at the municipality, sub-municipality and community levels. Each sub-municipality is further divided into communities for purposes of organizing Child Welfare Services (CWS) and other health related activities. The administrative division actually enabled the author to randomly select communities for the purpose of the study. It also ensured that the sample selected was a true representation of the entire municipality thereby avoiding bias.

Child Welfare Services are organized once every month in each of the communities. The services are part of the interventions under the Reproductive and Child Health Program in Ghana.

This study is a community based cross-sectional study which employs quantitative and qualitative method of data collection. The study involved 260 mother-infant pairs attending the community-based Child Welfare Clinic organized by the Municipal Reproductive and Child Health (RCH) Unit during the study period. Out of the 260 mother-infant pairs who participated in the study, 225 mother-infant pairs with infant up to seven months of age attending child welfare clinics within the communities were selected for the quantitative aspect of the study. The remaining 35 mother-infant pairs were selected and used for the qualitative aspect of the study.

Two communities were selected at random from each of the four sub-municipalities. This was done by writing the name of each sub-municipality on a box. Sheets of papers labeled with the names of communities within each sub-municipality were folded and put in their respective boxes. Community Health Nurses (CHN’S) were asked to pick two communities from each box. To ensure that each community has equal chance of being selected, the first paper (community) that was selected was replaced and reshuffled before selecting the second community. This step was repeated until the author was able to select two communities each from the four sub-municipalities.

Universal sampling technique was used in selecting the eligible mother-infant pairs who attended the Child Welfare Clinic during the study period. Data for the study was collected according to the CWC schedule. This was obtained from the health facilities within the various sub-municipalities.

After obtaining verbal consent from eligible mothers, face-to-face structured interviews were conducted using a pretested questionnaire during mothers visit to CWC within the study period. The questionnaire was pretested among 15 mother-infant pairs from a health facility (RCH unit) within the municipality. A 24 h self-recall method was used for assessing exclusive breastfeeding and breastfeeding frequency of mothers. Two volunteer CHN’s were trained on the administration of the questionnaire to assist the author in the data collection. Data were collected by Community Health Nurses were done under the supervision of a Registered Nurse.

Data on maternal sociodemographic variables (maternal age, education, religion, place of residence, sex and age of infant) and maternal work features (sector of employment, type of occupation, work location, risks of mother’s work on infant health, going to work with the infant, number of hours worked and distance from home to workplace) were collected. Data were collected once from each CWC to avoid interviewing the same mother-infant pair more than once during the study period. Mothers who visited the CWC with children of their relatives were excluded from the study.

The qualitative data were collected through the use of field notes. Focus group discussions were organized to discuss the results of the quantitative data collected at CWC in the communities. One CWC was selected for the focus group discussions because it has the highest attendance within the municipality. The clinic appointment gave author the opportunity to meet the same mother-infant pairs who participated in the face-to-face interview. Interviews and discussions were conducted in Fante, a dialect of Akan, since that is the Lingua-franca of the people of Effutu. Questions were prepared in English but were translated into Fante for mothers during the discussion.

The focus group discussion was facilitated by the author. Verbal consent was obtained from all mothers who participated in the discussion. This was done by explaining to mothers the aims of the focus group discussion and requesting individual mothers who wished to participate in the discussion to meet with the author after they have been attended to by nurses at the clinic. The 35 mother-infant pairs selected for the discussion were divided into three groups. The average number of mother-infant pair in each group was 12. The rules of the discussion were explained to mothers before the discussion and individual participants were not allowed to dominate the discussions. Answers to questions and contributions were taken in turns by mothers.

Discussions centered on the following: 1) How mothers managed to exclusively breastfeed their infants irrespective of demands of their work. 2) Reasons that accounted for mothers’ ability to breastfeed many times in a day. 3) Features of the mothers’ work that encourage or discourage breastfeeding in the manner taught by health professionals. 4) General issues within mothers’ environment that negatively or positively affect exclusive breastfeeding.

Data in the questionnaires were coded and entered into Statistical Package for Social Science (SPSS) version 20.0. The dependent variables were exclusive breastfeeding and breastfeeding frequency. Frequencies and cross-tabulations were run and a Chi-square test, specifically Fisher’s Exact Test, was used to identify strength of association between features of maternal work and exclusive breastfeeding practice. A *p* - value < 0.05 was used as the criterion for statistical significance.

The qualitative data were transcribed from Fante into English language. The field notes were taken by a research assistant proficient in both the English and Fante languages. The field notes were transcribed into English text by the same research assistant at the end of the discussion. The transcribed data from the focus group discussions were perused, categorized and summarized by the author and triangulated with the quantitative data.

### Definition of variables

Exclusive breastfeeding: This is defined as infant 0 to 6 months who were fed with breast milk without any other liquid or solid except drops and syrups (vitamin, minerals and medicines) in the previous 24 h.

Breastfeeding frequency: This is defined as the number of times infants were breastfed in the previous 24 h. This was coded based on the recommended number of times infants should be nursed per day [4, 5]. This is coded ‘1’ if a mother nursed 8 + times per day and ‘0’ if she nursed below 8 times per day.

Timely initiation of breastfeeding: This is defined as putting the newborn baby to the breast within the first hour of birth.

Maternal work: This refers to economic activities of mothers either on full-time or part-time to earn a living or support the home. In this study, mothers were given the option to indicate the actual economic activities they under take to earn a living or to support their homes.

Features of maternal work: This refers to the characteristics of mother’s work and includes the type of occupation, the sector of employment, place of work, conditions at work place, risks posed by mother’s work to infant health (examples of risks include smoke, heat from the sun, harmful objects and substances), mother working with her infant, distance from home to work place and the work location.

Formal employment: This refers to paid jobs and/or situations where mothers work in formal organizations such as schools, hospitals, banks, factories and supermarkets.

Informal employment: This refers to mothers who are self-employed; usually in small enterprise such as, subsistence farming, dress making, hair dressing, trading, catering and other forms of self-employment.

## Results

Table 1 shows the sociodemographic characteristics and exclusive breastfeeding practice of respondents. From a total of 225 mother- infant pairs that were interviewed, 200 were included in the analysis, giving a response rate of 89%. The remaining 25 respondents had babies older than seven months and did not meet the inclusion criteria. The mean (± SD) age of mothers and infants were 27 (±7) years and 4.3 (±2.3) months respectively.

**Table 1:** Sociodemographic characteristics and breastfeeding practice of mothers in Effutu Municipal, Central Region of Ghana, July to November, 2015 (*n* = 200)

Sociodemographic variables	Number	Percentage (%)
Age of mother (years)		
15 – 25	98	48.8
26 – 35	67	33.3
36 – 45	28	13.9
46 +	7	4.0
Mean age in years	28 ± 7	
Marital status		
Single	40	20
Married	160	80
Religion		
Christianity	188	94
Islam	5	2.5
Traditional religion	2	1
Other religion	5	2.5
Maternal education		
No education	65	32.3
Basic education	100	50.2
Secondary education	14	7.0
Tertiary education	21	10.4
Place of residence		
Slum	120	60
Planned community	80	40
Age of infant		
0 – 2 months	46	23.4
3 – 4 months	60	29.8
5 – 6 months	35	17.4
7 months	59	29.4
Mean age in months	4 ± 2	
Sex of infant		
Male	104	51.7
Female	96	48.3
Occupation		
Mothers without occupation	28	14
Mothers in occupation	172	86
Sector of employment		
Formal sector	22	13
Informal sector	150	87
Breastfeeding practice	Number	Percentage (%)
Breastfeeding initiation		
Within 1 h of birth	132	66
2 – 6 h	35	18
7 – 12 h	8	4
1 day and beyond	25	12
Exclusive breastfeeding by age of infant		
0 – 2 months	40	35
3 – 4 months	43	40
5 months	25	25
Breastfeeding frequency (8 +) in 24 h by age of infant.		
0 – 2 months	36	23
3 – 4 months	45	29
5 – 6 months	30	20
7 months	44	28
Breastfeeding frequency (<8) in 24 h by age of infant.		
0 – 2 months	11	24
3 – 4 months	14	32
5 – 6 months	5	11
7 months	15	33

**Table 2:** Exclusive breastfeeding and features of maternal work using 24 h self-recall

Features of maternal work	Exclusive breastfeeding				*p* - value
	Yes	%	No	%	
Type of occupation					
No occupation	16	11	7	15	
Beauticians	5	3	3	6	
Dress making	14	9	2	4	
Hawking trading	10	7	3	6	
Trading	50	35	17	36	
Fish mongering	27	18	11	25	
Teaching	15	10	2	4	
Catering	6	4	2	4	
Banking	2	1	0	0	
Nursing	3	2	0	0	
	Chi-square = 6.026				0.737
Work related risk					
Risky to health of infant	69	66	25	78	
Not risky to health of infant	35	34	7	22	
	Chi-square = 1.591				0.148
Sector of employment					
Formal sector	21	16	1	3	
Informal sector	112	84	38	97	
	Chi-square = 4.729				0.020
Work location					
Home	59	48	18	44	
Away from home	64	52	23	56	
	Chi-square = 0.204				0.163
Go to work with infant					
Do go to work with infant	64	57	17	52	
Do not go to work with infant	49	43	16	48	
	Chi-square = 0.271				0.373
Distance from home to work place					
Quarter – half km	17	24	3	20	
Half – one km	20	28	1	7	
One – two km	25	35	7	47	
Two or more km	10	13	4	26	
	Chi-square = 4.104				0.251
Working hours per day					
6 h	44	51	20	69	
8 h	33	38	5	17	
12 or more hours	10	11	4	14	
	Chi-square = 5.933				0.115

Among the mother infant pairs interviewed, 48.8% were between the ages of 15 – 25. The greater proportion (50.2%) of respondents had a basic education. Among those interviewed 60% are slum dwellers. Christianity is the dominant religion (94%). The majority (80%) of mothers are married. The number of male infants (51.7%) was slightly higher than female (48.3%). Almost all (100%) of the mothers in the study were breastfeeding at the time of the study.

Out of the 199 mothers who were breastfeeding at the time of the survey, the exclusive breastfeeding rate of an infant ≤ 5 months measured by the 24 h recall period preceding the survey, was 72%. The mean number of breastfeeding frequency per day was 11 ± 2.7 and the median breastfeeding frequency per day was 13.5.

Breastfeeding initiation was also universal. Mothers who initiated breastfeeding within the first hour of birth formed 67% of the total number of mother-infant pair interviewed. Further interviews identified that late initiation of breastfeeding was due to no milk in the mother’s breast and assisted delivery (specifically caesarean section). Regarding mothers who introduced supplementary foods, 53% introduced water and 23% introduced water and infant formula.

Responses from mothers during the focus group discussions indicated that some mothers still practiced exclusive breastfeeding even at seven months of age. These mothers mentioned that they had not yet introduced their infants to complementary feeds because they had no knowledge of the type of food to give them.

Table 2 provides information on the association between the features of maternal work and the exclusive breastfeeding rate. Eighty four percent of mothers in the informal sector of employment practiced exclusive breastfeeding compared to their counterpart in the formal sector of sector of employment (16%), (*p* = 0.020). The focus group discussions investigated how the majority of respondents in the study practiced exclusive breastfeeding and they identified flexibility of maternal work, scheduled feeding and family support as the major factors that explain mothers’ ability to exclusively breastfeed from 0 to 6 months without interference from their work.

**Table 3:** Breastfeeding frequency and features of maternal work using 24 h self-recall

Features of maternal work	Exclusive breastfeeding				*p* - value
	Yes	%	No	%	
Type of occupation					
No occupation	20	13	3	7	
Beauticians	5	3	3	7	
Dress making	12	8	4	9	
Hawking trading	12	8	1	2	
Trading	57	38	10	22	
Fish mongering	30	20	8	17	
Teaching	7	6	10	22	
Catering	4	3	4	8	
Banking	0	0	2	4	
Nursing	2	1	1	2	
	Chi-square = 28.485				0.001
Work related risk					
Risky to health of infant	73	78	23	62	
Not risky to health of infant	21	22	14	38	
	Chi-square = 1.835				0.127
Sector of employment					
Formal sector	12	9	10	23	
Informal sector	117	91	33	77	
	Chi-square = 5.629				0.021
Work location					
Home	59	48	18	44	
Away from home	64	52	23	56	
	Chi-square = 0.204				0.394
Go to work with infant					
Do go to work with infant	66	64	13	32	
Do not go to work with infant	37	36	28	68	
	Chi-square = 13.043				0.000
Distance from home to work place					
Quarter – half km	13	22	7	25	
Half – one km	12	20	9	32	
One – two km	21	36	11	39	
Two or more km	13	22	1	4	
	Chi-square = 5.261				0.154
Working hours per day					
6 h	43	51	21	66	
8 h	30	36	8	25	
12 or more hours	11	13	3	9	
	Chi-square = 2.224				0.527

It was mentioned that mothers in an occupation such as fish mongering, hawking trade and other trading activities, do not go to work with their children due to the risk (smoke and heat from the scorching sun) it poses to the health of their infants. These women had the flexibility to leave their infants in the care of other relatives and older siblings and to visit the house at frequent intervals to breastfeed their babies. A few also left their infants with nursing relatives who nurse their babies on their behalf until they returned home after work.

Table 3 contains the results of the features of maternal work and breastfeeding frequency. The findings of the chi-square test established a significant difference in breastfeeding frequency (No occupation 13%, beauticians 3%, dress making 8%, hawking trading 8%, trading 38%, fish mongering 20%, teaching 6%, catering 3% and nursing 1%) among mothers in the different occupation in the study (*p* = 0.001). More mothers (91%) in the informal sector of employment breastfed eight or more times in a day compared to those in the formal sector of employment (9%) (*p* = 0.021). It was also found that mothers who go to work with their infants (64%) breastfeed eight or more times in a day compared to those that do not (36%) (*p* = 0.000).

It came out during the focus group discussions that it was possible for most mothers to breastfeed more than eight times per day because of the practice of bed sharing during the night. Almost all of the participants of the focus group discussions mentioned that they share same bed with their infant during the night. They were able to breastfeed more during the night not only because they practiced bed sharing; but also due to the belief that it is a bad omen for infants to cry in the night. They mentioned that evil forces are believed to engage in nocturnal activities, and children who cry in the night can attract spells from evil forces. They therefore pay more attention to their infant during the night and pacify them with breast milk each time they attempt to cry.

## Discussion

Exclusive breastfeeding for the first six months of life provide the full nutritional requirements for healthy term infants. Continued breastfeeding when complementary foods are added, provide the essential part of child nutrition into the second year and beyond [2]. This study aims to investigate the association between the features of maternal work and exclusive breastfeeding practice.

Exclusive breastfeeding rate of infant ≤ 5 months was 72%. This is higher compared to the national figure (62.8%) reported by the World Bank [23]. The mean number of breastfeeds in the previous 24 h was 11 ± 2.7and the median number of breastfeeds was 13.5. This is also higher compared to Southwestern Nigeria (9.7 ± 3.9) and Southeast Ethiopia (six times per day) [17, 24]. Mothers who initiated breastfeeding within the first hour of birth (67%) were consistent with the WHO recommended. This finding is also higher compared to previous studies conducted in South East Ethiopia, Sudan (54.2%) and Jordan (49.5%) [23, 25, 26].

Sector of employment may be an important predictor of exclusive breastfeeding. In this study, the majority of mothers working in the informal sector of employment practiced exclusive breastfeeding compared to their counterparts in the formal sector of employment. This result is quite similar to findings from the UK that part-time and self-employed mothers were more likely to breastfeed for longer period than those in full-time employment [27].

The significant difference in exclusive breastfeeding between mothers of formal and informal sectors of employment could be explained by the flexible nature of work of mothers in the informal sector, as revealed by the focus group discussions. This follows similar findings from Iran which found that mothers with a reasonably flexible work schedule expressed no difficulty in breastfeeding their infants [28]. A plethora of evidence also exists, to show that where adequate alternative childcare is available, maternal employment does not negatively affect breastfeeding [29–31].

The focus group discussions also emphasized the role of family support in exclusive breastfeeding practice. Support from relatives and older siblings with the care of the infant during working hours was among the factors that influence exclusive breastfeeding among mothers with an occupation such as hawking trading, fish mongering and trading in general. This finding is consistent with findings from Bangladesh and in high and middle-income countries. It was found that physical/instrumental support increased the duration of both exclusive and partial breastfeeding [26, 32].

However, the practice of allowing infants to be breastfed by nursing relatives may have its own disadvantages. It is said that certain infectious diseases such as HIV, untreated active tuberculosis, drugs and chemicals can be transmitted through breast milk [5, 33]. It is possible that this practice can promote the spread of infectious diseases from nursing mothers to other infants, especially in situations where a relative may have any of these diseases or is on illicit drugs and her condition is unknown to family members.

The significant difference established between breastfeeding frequency and maternal occupation (*p* = 0.001), the two sectors of employment (*p* = 0.021) and the practice of going to work with the infant (*p* = 0.000) had no literature available. Existing literature focuses on the mean number of breastfeeding per day and do not consider factors associated with breastfeeding frequency.

As revealed by the focus group discussions, infants were breastfed many more times in the night than during the day. This practice is consistent with a study conducted in Southwestern Nigeria, which found that breastfeeding a child at night is common [34]. The belief that the cry of infant during the night attract spells from evil forces is reinforced by a popular Ghanaian lullaby which is literally translated;

*Do not cry my little baby at night so that something does not take away your voice.* ‘Meaning if a child cries at night something bad can happen to him or her’.

According to some oral traditions in Ghana, this belief is significant in the sense that it provides couples and their babies as well as neighbors with the following benefits: 1) Mothers attempt to cuddle and pacify babies with breastfeeds in the night provides babies with the mother-child attachment that they enjoyed during the gestational period, thereby building the bond between mother and child. 2) Mothers forgoing their sleep to breastfeed and calming babies at night portray the love they admit to have for their babies. 3) It helps society to differentiate between a “good” and a “bad” mother. It is believed that the ‘good’ mother is one that is able to sacrifice her pleasure for the benefit of her children. 4) It promotes a peaceful co-existence between the mother and her neighbor by ensuring that babies do not cry at night to disturb neighbors. 5) Pacifying babies with breastfeeding at night prevents babies from interfering with love making which is believed to usually occur at night in Ghana.

This study has a number of potential limitations. Information on exclusive breastfeeding and breastfeeding frequency was based on 24 h recall and does not represent the proportion of infants that were breastfed in the manner described by the result and should not be interpreted as such. The study is subject to information biases because the interviews were done in the presence of Community Health Nurses who are charged with the responsibility to promote appropriate infant and young child feeding practices at Child Welfare Clinics.

Even though verbal consent was obtained from respondents and participation in the study was not compulsory, almost all eligible mothers visiting the CWC on the day of the interview agreed to participate in the study. It is possible that they agreed to participate in the study because of the presence of the CHN’s at the clinic. The study also uses a cross-sectional study design, which makes it difficult to establish causal associations.

## Conclusion

The study reveals that timing of the initiation of breastfeeding and exclusive breastfeeding frequency follows WHO recommendations and the Ghana breastfeeding policy. More mothers in the informal sector of employment exclusively breastfeed their infants and breastfeed more than eight times in the previous 24 h compared to mothers in the formal sector of employment. Family support, bed-sharing, flexible work schedule and cultural beliefs are key factors of exclusive breastfeeding and breastfeeding frequency. Interventions for improving exclusive breastfeeding practice among working mothers should include reliance on existing family structures which promote exclusive breastfeeding. It is also important that we identify cultural believes and practices that support infant and young child feeding in each society and use them to promote exclusive breastfeeding. Policy makers should consider promotion of infant-friendly work environment among employers and the establishment of work-site crèches to promote exclusive breastfeeding.
